# Enhancing Ligand Epitope Mapping in Biomolecular Recognition by Variable Temperature Saturation Transfer Difference NMR (VT‐STD NMR)

**DOI:** 10.1002/mrc.70141

**Published:** 2026-09-16

**Authors:** Gabriel Rocha, Svetla Daskalova, Juan C. Muñoz‐García, Jesús Angulo

**Affiliations:** ^1^ Biomolecular Interactions and Structural Glycobiology Group Institute for Chemical Research (IIQ), CSIC – University of Seville Seville Spain; ^2^ Faculty of Chemistry and Pharmacy University of Würzburg Würzburg Germany

## Abstract

Saturation transfer difference nuclear magnetic resonance (STD NMR) spectroscopy is a powerful and robust technique for the characterization of receptor–ligand interactions of medium to weak affinity in solution, providing binding epitope maps that reveal structural details of these interactions at atomic level. This knowledge is essential for understanding the structural basis of biomolecular recognition processes of biological relevance and plays a key role in drug discovery. Temperature is a critical factor influencing the sensitivity of STD NMR spectroscopy, as it directly affects the kinetics of complex formation, thereby modulating the observed STD effects. In this study, we systematically investigated the impact of temperature on STD NMR‐derived binding epitope maps. Our findings highlight that, whenever protein stability permits, temperature should be considered an essential experimental parameter to be optimized for each system to obtain binding epitope maps with well‐discriminated protein–ligand contact information. For the example cases studied herein, we demonstrate that temperature optimization allows to better assess the contribution of each individual contact to protein–ligand binding (enhanced ligand epitope mapping discrimination).

## Graphical abstract

The impact of temperature on the analysis of STD NMR ligand epitope mapping has been thoroughly investigated. For protein–ligand complexes under fast exchange kinetics, a negligible temperature effect is observed. For the intermediate exchange regime, VT‐STD NMR allows to obtain better discriminated epitope mappings, hence enabling the accurate determination of the main “hot spots” of molecular recognition.

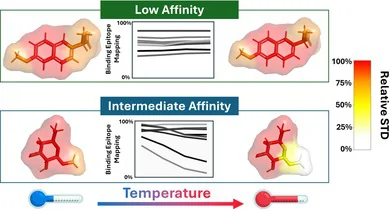

## 1 Introduction

The specific interactions between molecules in solution through non‐covalent forces play a fundamental role in the chemistry of molecular recognition. Over the past decades, nuclear magnetic resonance (NMR) has proven to be an invaluable tool for elucidating the atomic‐level structure of these processes in solution [ 1, 2, 3 ] . Numerous NMR experiments have been developed and successfully applied to a wide range of systems, particularly those involving biologically relevant receptor–ligand interactions [ 4, 5, 6, 7 ] . Among ligand‐based NMR techniques, saturation transfer difference (STD) NMR spectroscopy has emerged as one of the most powerful and versatile methods, with growing applications in both academic research and the pharmaceutical industry, particularly in drug discovery, ligand design, and optimization [ 8, 9, 10, 11, 12 ] .

The magnitude of the STD NMR response is highly sensitive to the fraction of protein–ligand complexes present in solution, which is governed by the dissociation constant (K_D_ ) that determines the equilibrium distribution between bound and free species. This fraction can be modulated by adjusting protein and ligand concentrations as well as the working temperature [ 13 ] . Since the STD NMR experiment is based on a difference spectroscopy approach, the resulting spectrum exclusively contains ligand signals, which bear direct information about the concentration of the protein–ligand complex [ 14 ] . Consequently, STD NMR spectroscopy is a valuable method for determining K_D_ through titration experiments, fitting the experimental STD NMR data to a Langmuir isotherm [ 11, 15, 16, 17 ] .

(1) $$K_{D}=\frac{\left[P\right]\left[L\right]}{\left[\mathrm{PL}\right]}=\frac{k_{\mathrm{off}}}{k_{\mathrm{on}}}$$

The average duration of the receptor–ligand complex formation depends on both the association rate (k_on_ ) and the dissociation rate of the complex (k_off_ ), and both are affected by temperature. The dissociation rate reflects the ligand's residence time in the complex in solution (1/k_off_ ) [ 18 ] .

In 2001, Jayalakshmi and Krishna proposed that ligand signal intensity variations in STD NMR experiments are primarily governed by six parameters: (1) saturation time, (2) spatial proximity of receptor‐saturated protons relative to ligand protons, (3) ligand–receptor interface conformation, (4) ligand‐to‐receptor ratio, (5) rotational correlation times of both receptor and ligand (and hence, T_1_ and T_2_ relaxation times), and (6) kinetics of reversible complex formation [ 19 ] . Since the last two parameters are strongly affected by temperature, it follows that temperature is a critical parameter affecting the observed STD intensities due to its impact in the residence time of the ligand in the bound state. Previously, a study by Jiménez‐Barbero and co‐workers analyzed the effect of temperature on STD NMR intensities, showing that the kinetics and thermodynamics properties of binding determine the direction of intensity change, in an unpredictable way, so that they can increase, decrease, or even remain constant with temperature [ 20 ] . However, the impact of temperature on the discrimination capacity of STD NMR binding epitope mappings and DEEP‐STD NMR data is yet to be thoroughly investigated.

The dependence of STD NMR on the existence of a fast chemical exchange process makes this technique ideally suited for low‐to‐medium affinity interactions, i.e., 1 μM < K_D_ < 1 mM approximately. For high affinity ligand binding (K_D_ < 1 μM), the ligand's residence time in the bound state is generally long (within the second/s range) and, hence, during the protein saturation time, minimal ligand exchange will occur between the bound and free states. As a result, no intensity saturation accumulates in solution, leading to an absence of detectable STD NMR signals. Conversely, if the residence time is too short (K_D_ > > 10’s of mM, low affinity) chemical exchange is too fast, preventing effective protein–ligand NOE transfer to occur and, again, no STD intensities are observed. Since the rate of chemical exchange is affected by temperature, optimizing this experimental parameter allow, in favorable cases, detection of ligand binding falling in the low or high affinity STD NMR limits by reducing or increasing, slowing down or accelerating the chemical exchange process, respectively.

The magnitude of the observed STD NMR signals is not only dependent on the spatial proximity of ligand protons to the receptor and the kinetics of exchange, but also by the different longitudinal relaxation times (T₁) of ligand protons, which depend on their correlation times (a proxy for local proton mobility) and, thus, on temperature [ 21 ] . The latter can lead to misleading structural information on the main protein–ligand contacts. To circumvent this issue, Mayer and James proposed to record STD NMR build‐up curves for each ligand proton and derive their initial slopes through mathematical fitting to a mono‐exponential equation ( Equation 2 ) [ 22 ] .

(2) $$\mathrm{STD}\left(t\right)={\mathrm{STD}}_{\mathrm{MAX}}\left(1-e^{\left(-k_{\mathrm{SAT}}\cdot t\right)}\right)$$

where STD (*t*) represents the intensity of the STD signal of a proton as a function of saturation time (*t*), STD_MAX_ is the maximum STD intensity determined at very long saturation times, and k_SAT_ represents the observed saturation rate constant and depends on the longitudinal relaxation and binding kinetics. The initial growth rate of the STD build‐up curve (STD_0_) is defined as follows:

(3) $${\mathrm{STD}}_{0}=\lim_{t_{\mathrm{SAT}}\to 0}\frac{\text{δSTD}\left(t_{\mathrm{SAT}}\right)}{\delta t_{\mathrm{SAT}}}={\mathrm{STD}}_{\mathrm{MAX}}\cdot k_{\mathrm{SAT}}$$

By normalizing the STD_0_ values of the individual ligand protons against the highest one (to which an arbitrary value of 100% is assigned), the relative STD factors (STD_rel_) are obtained. These are used to generate the so‐called STD NMR ligand epitope mapping.

It is also noticeable that spin diffusion within the network of protons of the protein does not always guarantee homogeneous receptor saturation, so differences in ligand binding epitope maps may arise when STDs are acquired at different irradiation frequencies. As the frequencies of irradiation are known, those epitope variations can be exploited to gain insights into the type of protein residues (aromatic, aliphatic, polar, or apolar) interacting with the ligand protons, yielding a differential ligand binding epitope map that provides information of the protein's pharmacophoric features. This differential epitope mapping technique is known as DEEP‐STD NMR [ 23 ] . As the spin diffusion mechanism operates at the limit of the negative NOE regime, it is more efficient for very slowly tumbling molecules. Temperature plays a crucial role in regulating molecular motion and hence optimizing it may allow to reach a non‐homogeneous spin diffusion within the receptor, and this is expected to increase the discrimination capacity of differential epitope mapping measurements.

Here, we have carried out a systematic analysis of the effect of temperature on STD NMR and DEEP‐STD NMR experiments, strongly recommending the use of variable temperature STD NMR (VT STD NMR) to improve the insights into protein–ligand recognition that is gained from STD NMR measurements. To do so, we have employed a medium and a low affinity protein–ligand complex as model systems.

## 2 Results and Discussion

We have systematically investigated the effect of temperature on the various parameters influencing STD NMR intensities for two protein–ligand complexes showing different kinetic regimes. The first is the complex between the plant lectin agglutinin 120 from *Ricinus Communis* Agglutinin I (RCA120) and the ligand methyl‐*β*‐D‐galactoside (*β*‐Gal‐OMe; see ESI, Figure S1 ), for which dissociation constants (K_D_ ) of 260 μM and 283 μM have been reported by calorimetry [ 24 ] (20 °C) and STD NMR titration experiments [ 17 ] (25 °C), respectively, placing it in the medium‐to‐high affinity or slow exchange regime.

The other system studied is the complex formed by bovine serum albumin (BSA) and the ligand Naproxen (NPX; see ESI, Figure S1 ), for which the dissociation constant was determined using the Reduce Dataset (rd‐STD NMR) approach, yielding an apparent K_D_ of 914 μM at 5 °C (see ESI, Figure S2 ). It should be noted that BSA contains multiple binding sites for naproxen, including sites of substantially higher affinity than the apparent K_D_ determined here by rd‐STD NMR. Since STD NMR relies on continuous exchange between free and bound ligand molecules, the experimentally observed STD NMR signals predominantly originate from the exchanging ligand population contributing to saturation transfer. Therefore, the apparent affinity obtained from STD NMR measurements should not be interpreted as the intrinsic affinity of the highest‐affinity naproxen binding site on albumin, but rather as an effective parameter describing the binding events of appropriate kinetics to STD NMR detection under the experimental conditions employed. The purpose of including the BSA/NPX system in this study is not to establish the absolute binding affinity, or binding‐site distribution, or site‐specific epitope map, of naproxen binding to albumin, but to provide an example of a system exhibiting STD NMR behavior characteristic of the fast‐exchange regime and to assess how temperature affects the resulting STD observables. Although multiple binding sites may contribute to the observed STD signals, this heterogeneity does not affect the central objective of the present study, namely the assessment of how temperature influences STD_0_ , STD_MAX_ , k_SAT_ , binding epitope discrimination, and DEEP‐STD NMR observables.

These systems were selected due to its robust nature, high sensitivity, and high spectral dispersion of the ligand. For the first system (RCA120/*β*‐Gal‐OMe), a protein concentration of 20 μM (40 μM per binding site) and a ligand concentration of 4 mM were used, whereas for the second system (BSA/NPX), a protein concentration of 10 μM and a ligand concentration of 1 mM were employed, resulting in a 1:100 protein‐to‐ligand ratio in both cases. The STD NMR build‐up curves were conducted under identical conditions, except the temperature that was varied over the range of 5 °C–35 °C (see ESI, Figures S3–S10 ).

### 2.1 Temperature dependence of binding epitope maps

Since the two systems fall within different kinetic regimes, they are expected to respond differently to temperature variations. Accordingly, Figure 1 illustrates how the binding epitope maps obtained for both systems vary as a function of temperature. For the first system ( Figure 1 a ), an increase in temperature resulted in enhanced discrimination within the binding epitope map, enabling the extraction of more clearly defined structural information about the complex. In contrast, for the second system ( Figure 1 b ), no temperature‐dependent variation in the epitope map was observed.

**FIGURE 1:**
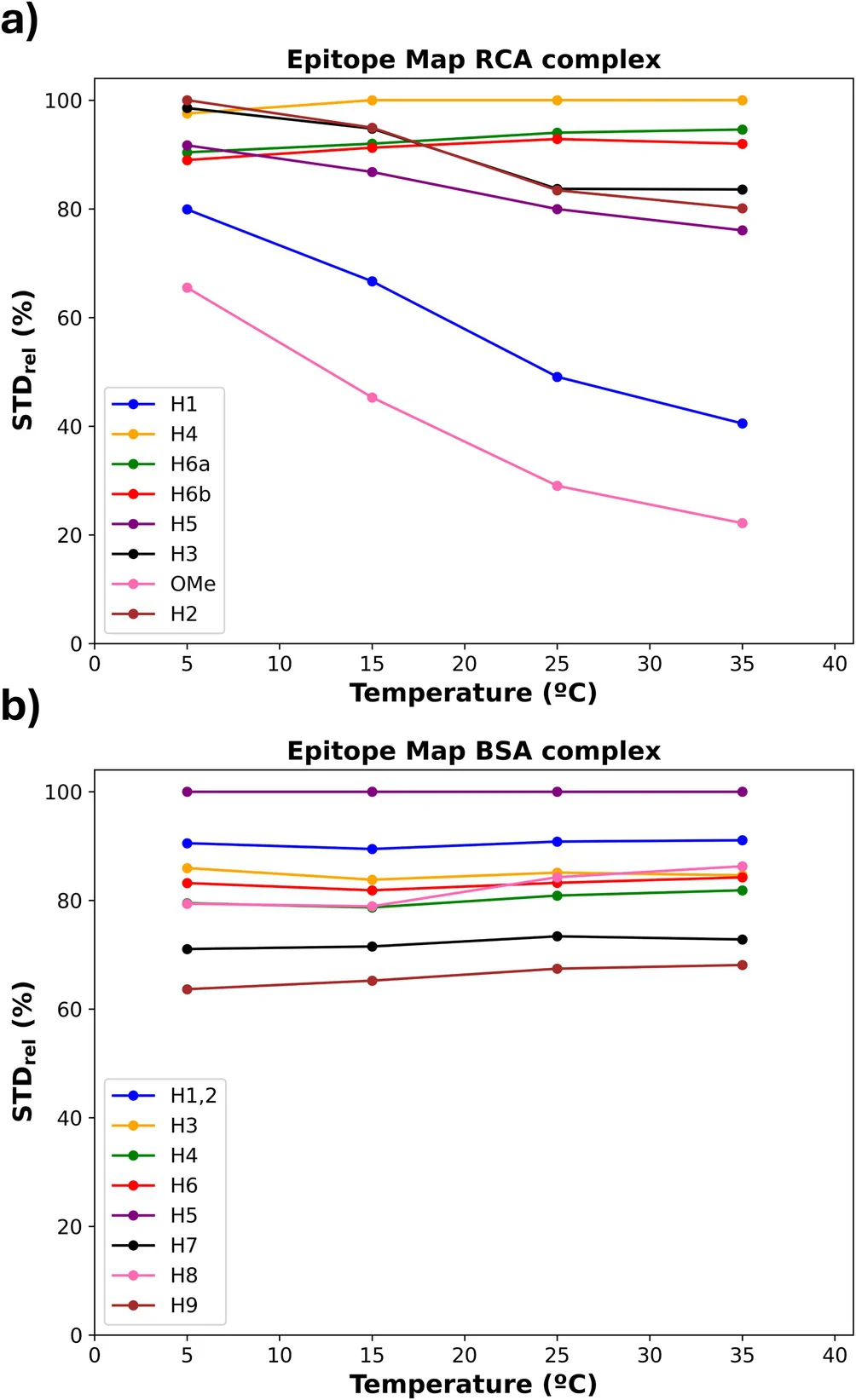
(a) Values of STD_REL_ (which determines the binding epitope map) for each proton of *β*‐Gal‐OMe as a function of temperature. (b) Values of STD_REL_ for each proton of NPX as a function of temperature.

The results suggest that, for systems operating in the slow‐exchange regime, in cases where the epitope is poorly defined (highly homogeneous ligand epitope map), an efficient strategy could be to increase the temperature to reduce the contribution of intramolecular spin diffusion in the bound ligand (more efficient at lower temperatures). In such cases, it would be necessary to ensure protein stability at the chosen temperature. In scenarios where only a decrease in temperature is practically feasible (due to protein instability), a non‐optimally discriminated ligand binding epitope mapping could be expected. While the “hot spots” of binding might still be identified under these conditions, their discrimination (or dynamic range observed between strong or weak contact among different ligand moieties) might be compromised.

In contrast, for systems in the fast‐exchange regime, temperature does not significantly influence the binding epitope map discrimination, as intramolecular ligand spin diffusion is negligible so that almost identical binding epitope maps are obtained across the accessible temperature range.

### 2.2 Temperature dependence of STD NMR initial slopes (STD_0_)

As shown above, the STD NMR binding epitope maps are affected by temperature for binding events within the slow exchange regime (RCA complex), as the STD_0_ values (used to obtain the binding epitope) are affected by temperature. For the RCA complex, the highest STD_0_ values of *β*‐Gal‐OMe protons were observed at 25 °C (most protons) or 15 °C (H1 and methyl protons) ( Figure 2 a ). This is due to the faster chemical exchange of the ligand at higher temperatures, resulting in greater accumulation of free saturated ligand in solution per unit of time. However, when temperature is further increased, chemical exchange becomes too rapid (reduced residence time in the bound state), reducing the efficiency of the global process of protein–ligand saturation transfer. As a result, STD₀ values started to decrease between 25 °C and 35 °C. This is due to the fact that the greater accumulation of saturated ligand at this higher temperature is surpassed by the loss of saturation transfer efficiency due to the much shorter bound state residence time. In other words, more saturated ligand molecules are accumulated in solution per unit of time, but bearing lower saturation levels, resulting in a net reduction of the observed STD values and STD_0_ .

**FIGURE 2:**
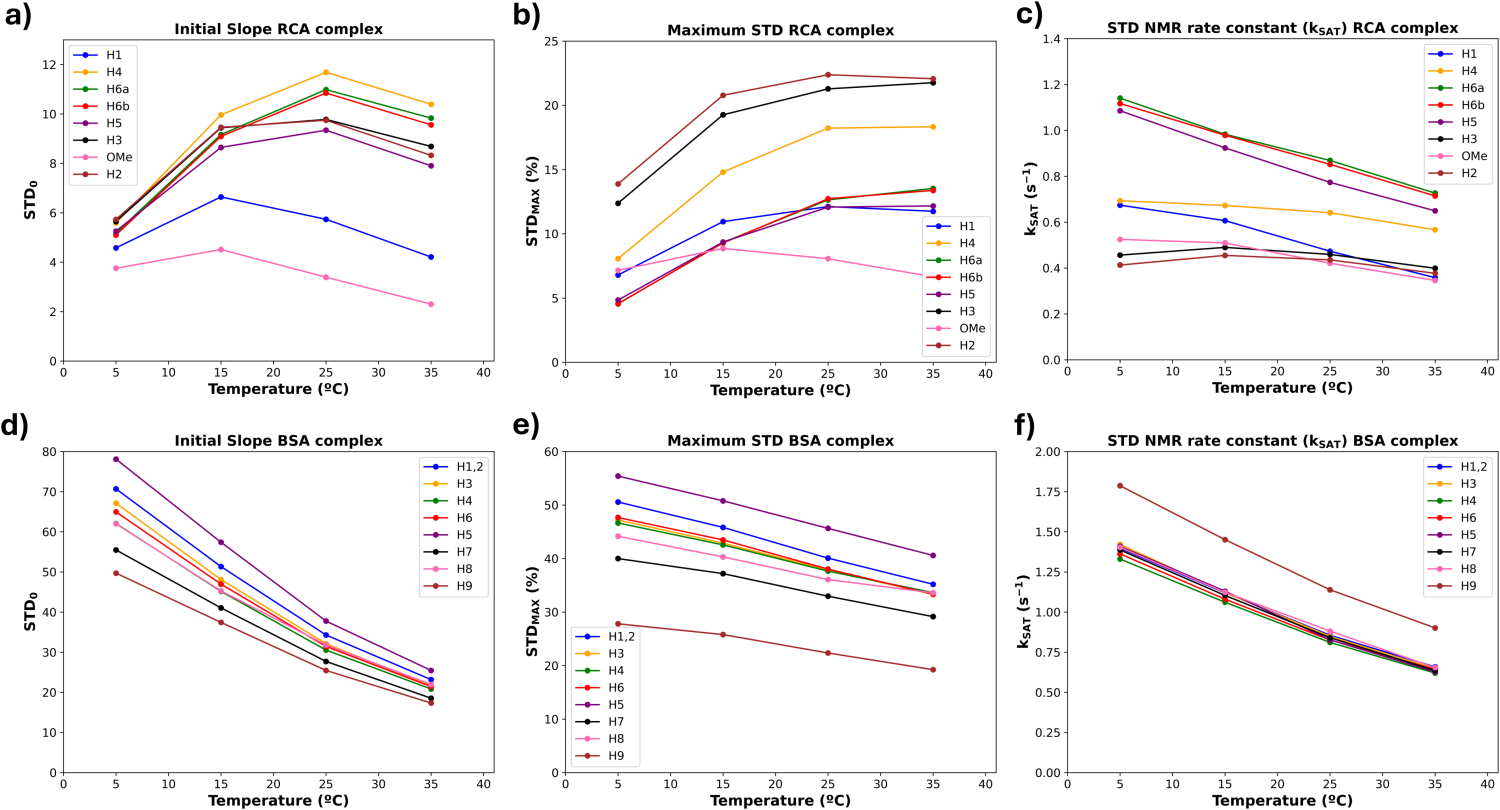
(a) Values of STD_0_ for each proton of *β*‐Gal‐OMe as a function of temperature. (b) Values of STD_MAX_ for each proton of *β*‐Gal‐OMe as a function of temperature. (c) Values of k_SAT_ for each proton of *β*‐Gal‐OMe as a function of temperature. (d) Values of STD_0_ for each proton of NPX as a function of temperature. (e) Values of STD_MAX_ for each proton of NPX as a function of temperature. (f) Values of k_SAT_ for each proton of NPX as a function of temperature.

Protons H_1_ and OMe showed the highest STD_0_ values at 15 °C, decreasing at higher temperatures. This is most likely due to being the most solvent exposed protons (see PDB structure 1RZO) and, thus, being more sensitive to the impact of temperature on ligand intramolecular spin diffusion. At lower temperatures, the ligand remains bound to the protein for a longer period so that each ligand proton receives greater saturation transfer from both the protein and the neighboring ligand protons (intramolecular NOE). This leads to the “homogenization” of the apparent saturation received by each proton (intramolecular binding epitope leakage). As the temperature increases, the reduced residence time of the bound ligand leads to lower intramolecular NOE contributions and, as a result, to a greater differentiation between STD₀ values ( Figure 2 a ). This results in better discriminated epitope maps ( Figure 1 a ).

In contrast, for the BSA complex (low affinity or fast exchange regime) a temperature increase leads to a decrease in STD₀. This is due to the faster kinetics of exchange of low affinity complexes resulting in low residence times of the bound ligand and, as a result, inefficient saturation transfer. Thus, when possible, it is generally preferred to work at lower temperatures for low affinity complexes.

### 2.3 Temperature dependence of STD_MAX_ and k_SAT_

The initial slope is the product of STD_MAX_ and k_SAT_ . STD_MAX_ represents the maximum saturation intensity transferred to a ligand proton. For systems in the intermediate‐to‐slow exchange regime ( Figure 2 b ), where the residence time (τ_res_ ) of the bound ligand is sufficiently long for efficient saturation transfer over the temperature range, an increase in temperature leads to an STD_MAX_ enhancement. This is due to the greater accumulation of free saturated ligand in solution as exchange kinetics increase with temperature. Conversely, in systems within the fast exchange regime ( Figure 2 e ), where the ligand τ_res_ is short enough to compromise saturation transfer efficiently, a temperature increase results in a decrease in STD_MAX_ (reduced saturation transfer efficiency overcomes the greater accumulation of saturated ligand).

Figure 2c,f shows the variation of the k_SAT_ values of each proton with temperature for both systems, showing a decreasing trend at higher temperatures. As the temperature increases, the Brownian rotational diffusion of the ligand in solution is accelerated, leading to less efficient interactions between spins and the magnetic field of neighboring nuclei. This results in an increase in the longitudinal relaxation time (T₁). Overall, the temperature‐dependent variation of k_SAT_ is more pronounced for protons that, due to the ligand molecular structure, exhibit highly efficient longitudinal relaxation. For instance, at lower temperatures the magnetic dipoles of the geminal protons H_6a_ and H_6b_, and H_5_, of *β*‐Gal‐OMe interact strongly, leading to faster relaxation rates and higher k_SAT_ values (Figure 2c). Consequently, their temperature variations are more significant compared to protons with less efficient longitudinal relaxation (e.g., H2, H3, H4 in Figure 2c). This is expected since the longer T_1_ times of the ligand at higher temperatures allow accumulation of saturated ligand for longer times, hence resulting in the decrease of k_SAT_. It is noticeable that the impact of temperature on k_SAT_ is more pronounced, overall, within the fast binding regime (BSA complex), where the increase of ligand T_1_ at higher temperatures correlates with lower accumulation of saturation in solution (Figure 2e), leading to a larger reduction of k_SAT_.

### 2.4 Analysis of the temperature dependence of differential epitope mapping (multifrequency or DEEP‐STD NMR)

Figure 3 displays the obtained values of the differential epitope mapping (aliphatic/aromatic) for the *β*‐OMe‐Gal ligand as a function of temperature. Positive ΔSTD values (H_6a_ , H_6b_ , and to a lesser extent H_5_ ) indicate a relative increase in STD upon irradiation on aliphatic residues, whereas negative ΔSTD values (H_1_ , H_2_ , and H_3_ ) indicate a relative increase in STD upon irradiation on aromatic residues. As the temperature increases, the magnitudes of the DEEP‐STD NMR factors also increase. In the case of H_4_ , it remains constant, reflecting a similar interaction with both types of residues. The impact on OMe protons appears to be random, as they are exposed to the solvent.

**FIGURE 3:**
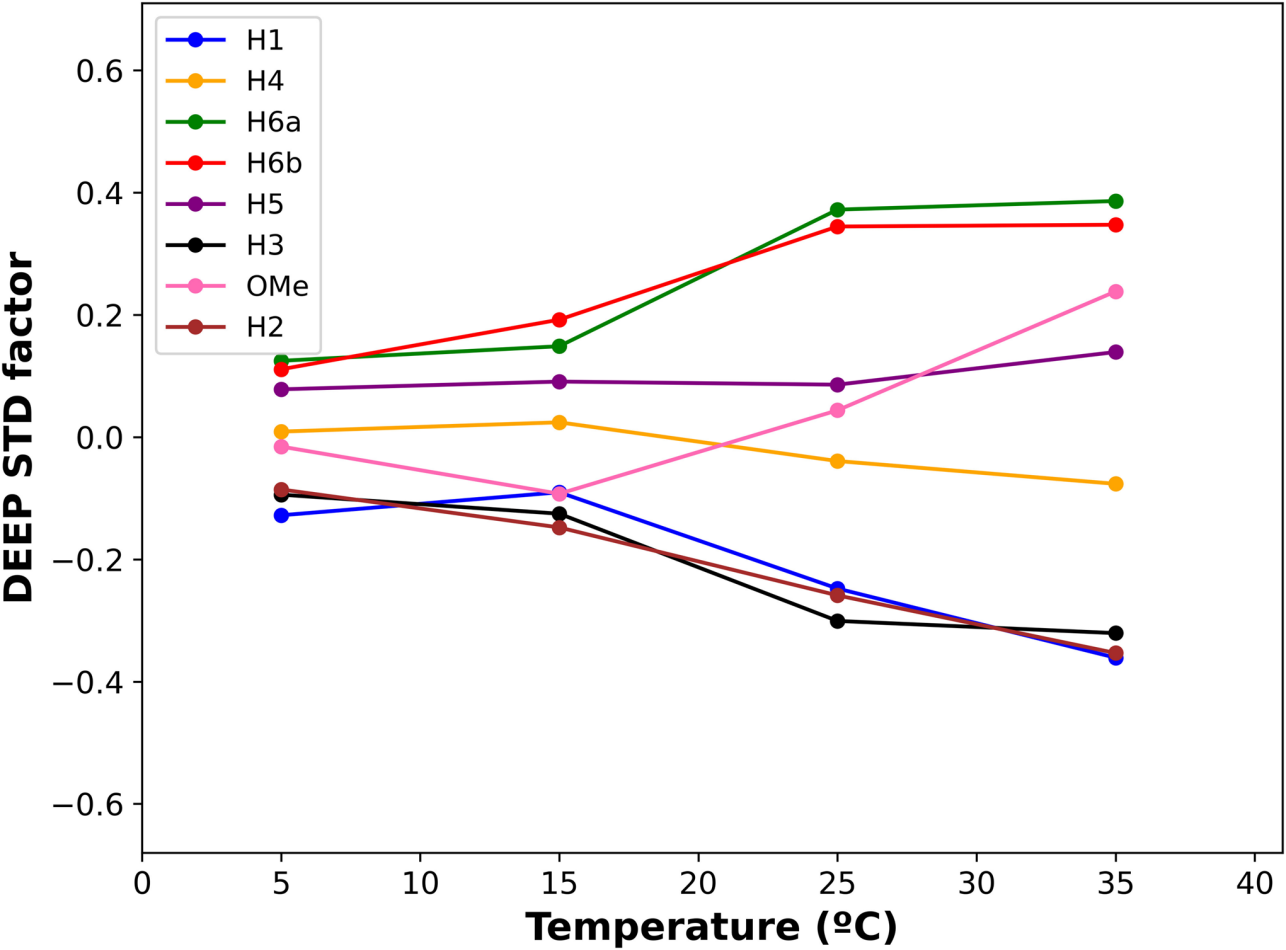
DEEP‐STD NMR values for each proton of *β*‐Gal‐OMe (RCA complex) as a function of temperature.

With increasing temperature, spin diffusion within the protein becomes less efficient. Therefore, upon irradiation of a specific region of the protein spectrum (e.g., aliphatic region), the saturation will be more efficiently transferred to the ligand protons that come into contact with irradiated residues, leading to a better discrimination between the *β*‐Gal‐OMe protons that orient towards aliphatic and aromatic regions of the protein binding site.

Finally, it should be noted that, as the DEEP‐STD NMR approach follows the same principles as the conventional STD NMR experiment, the complete relaxation and conformational exchange matrix theory for STD NMR (CORCEMA‐ST) allows to predict that the impact of temperature on DEEP‐STD NMR discrimination should be largely independent of the affinity, provided that the binding equilibrium remains within the fast exchange conditions required for the observation of STD NMR signals [ 19 ] . Under these conditions, DEEP‐STD NMR discrimination, in addition to its dependence on the architecture of the binding pocket, ultimately depends on the efficiency of intramolecular spin diffusion within the protein during saturation, which is determined by the protein's tumbling rate in solution and therefore influenced by factors such as protein molecular weight, working temperature, and solution viscosity.

## 3 Conclusions

In this study, a systematic investigation of the impact of temperature on all the parameters affecting and derived from STD NMR experiments has been carried out to assess how changes in binding kinetics and relaxation properties can affect the discriminative quality of the obtained binding epitope mappings. Two complexes operating in different kinetic regimes have been investigated: RCA120/*β*‐Gal‐OMe and BSA/NPX.

For the system under slower chemical exchange, the RCA120/*β*‐Gal‐OMe complex, increasing the temperature leads to an enhancement of STD initial slopes, and, at the same time, to a significantly enhanced discrimination between STD_0_ values, which allow for a better definition of the main ligand hot spots responsible for molecular recognition (binding epitope map), thereby providing more detailed structural information. However, an excessive temperature (e.g., over 35 °C) hinders efficient saturation transfer (reduced STD_0_ values). This is more dramatic in the case of fast exchange regime (BSA complex), where an increase in temperature systematically leads to reduced STD₀ values, while having no significant influence on epitope mapping.

Finally, we demonstrated that the reduction on spin diffusion efficiency within the protein upon increasing the temperature results in significantly better discrimination between DEEP‐STD NMR values, providing more precise information about the type ligand–protein contacts at the protein side chain level in multifrequency‐STD NMR experiments.

The results presented herein strongly support the use of VT‐STD NMR to enhance the discrimination of STD‐derived ligand epitope maps and DEEP‐STD NMR analyses. This is particularly recommended for new protein–ligand systems for which no structural information is available, and opens new avenues for a more reliable experimental validation of 3D molecular models of protein–ligand complexes.

## Author Contributions

**Gabriel Rocha:** methodology, validation, visualization, formal analysis, data curation, writing – original draft, investigation. **Svetla Daskalova:** formal analysis, visualization, validation, methodology, data curation. **Juan C. Muñoz‐García:** writing – original draft, conceptualization, writing – review and editing, project administration, supervision, methodology, investigation. **Jesús Angulo:** conceptualization, funding acquisition, writing – review and editing, writing – original draft, project administration, supervision, resources, methodology, investigation.

## Funding

This work was supported by Ministerio de Ciencia e Innovación, 10.13039/100014440, AEI/10.13039/501100011033/PID2022‐142879NB‐I00, and Erasmus+, 10.13039/501100010790.

## Supporting information


**Figure S1:** Chemical structures of methyl‐β‐D‐galactoside (left) and naproxen (right). Numbering is indicated with labels next to each carbon atom.
**Figure S2:** KD values obtained for each proton of the BSA complex using the rd‐STD NMR approach; 0.5 and 6 s were used as short and long saturation times, respectively.
**Figure S3:** Experimental STD NMR build‐up curves of RCA complex at 5 °C with irradiation at 0.66 ppm.
**Figure S4:** Experimental STD NMR build‐up curves of RCA complex at 15 °C with irradiation at 0.80 ppm.
**Figure S5:** Experimental STD NMR build‐up curves of RCA complex at 25 °C with irradiation at 0.90 ppm.
**Figure S6:** Experimental STD NMR build‐up curves of RCA complex at 35 °C with irradiation at 1.00 ppm.
**Figure S7:** Experimental STD NMR build‐up curves of BSA complex at 5 °C with irradiation at 0.00 ppm.
**Figure S8:** Experimental STD NMR build‐up curves of BSA complex at 15 °C with irradiation at 0.13 ppm.
**Figure S9:** Experimental STD NMR build‐up curves of BSA complex at 25 °C with irradiation at 0.25 ppm.
**Figure S10:** Experimental STD NMR build‐up curves of BSA complex at 35 °C with irradiation at 0.36 ppm.

## Data Availability

The data that support the findings of this study are available from the corresponding author upon reasonable request.
