# Supplementary material for: Enhancing Ligand Epitope Mapping in Biomolecular Recognition by Variable Temperature Saturation Transfer Difference NMR (VT‐STD NMR)
Source: Magn Reson Chem. 2026 Sep 16;64(12):1013–9. doi: 10.1002/mrc.70141 (PMC13725425; doi:10.1002/mrc.70141)
Supplement: Supplementary file 1 [file MRC-64-1013-s001.pdf]

Supporting Information

Enhancing Ligand Epitope Mapping in Biomolecular Recognition by Variable Temperature Saturation Transfer Difference NMR (VT-STD NMR)

Gabriel Rocha[a], Svetla Daskalova,[b] Juan C. Muñoz-García,*[a] Jesús Angulo*[a]

[a] G. Rocha, Dr Juan C. Muñoz-García, Dr J. Angulo
Biomolecular Interactions and Structural Glycobiology Group
Institute for Chemical Research (IIQ), CSIC – University of Seville
49 Américo Vespucio St, 41092, Seville, Spain
E mail: juan.munioz@iiq.csic.es, j.angulo@iiq.csic.es

[b] S. Daskalova
Faculty of Chemistry and Pharmacy, University of Würzburg
Am Hubland, 97074 Würzburg, Germany

TABLE OF CONTENTS

1. Materials and Methods **S2**
2. Ligand chemical structures **S2**
3. Dissociation constants (K_Ds) **S2**
4. STD NMR build-up curves **S3**

1. Materials and Methods

STD NMR experiments for the RCA complex were carried out using a Bruker Avance III 600 MHz spectrometer equipped with high-performance QCI quadrupole cryoprobe optimised for ^{1}H, ^{13}C, ^{15}N and ^{19}F detection. STD NMR experiments for the BSA complex were performed using a Bruker Avance III 700 MHz spectrometer equipped with a high-performance QCI quadrupole cryoprobe.

STD NMR experiments were carried out at 5, 15, 25 and 35 °C. The most shielded protein signal at each temperature was used as *on-resonance* frequency, this is, 0.66, 0.80, 0.90 and 1 ppm for the experiments acquired at 5, 15, 25 and 35 °C, respectively. The *off-resonance* frequency was set to 40 ppm. Varying saturation times ranging from 0.25 to 6 (BSA complex) or 8 s (RCA complex), a train of selective Gaussian saturation pulses of 50 ms, and a relaxation delay of 6.1 (BSA complex) and 8.1 s (RCA complex) were used. The residual protein resonances were filtered using a T1ρ filter of 25 ms. All STD NMR spectra were acquired using between 16 and 64 scans, with the lower and higher number of scans being used for the higher and lower saturation times, respectively. DEEP STD NMR experiments were acquired using the same *on-resonance* frequencies for the aliphatic irradiation as specified above, and aromatic irradiation frequencies of 6.77, 6.9, 7.0 and 7.1 ppm (for the experiments acquired at 5, 15, 25 and 35 °C, respectively).

2. Ligand chemical structures

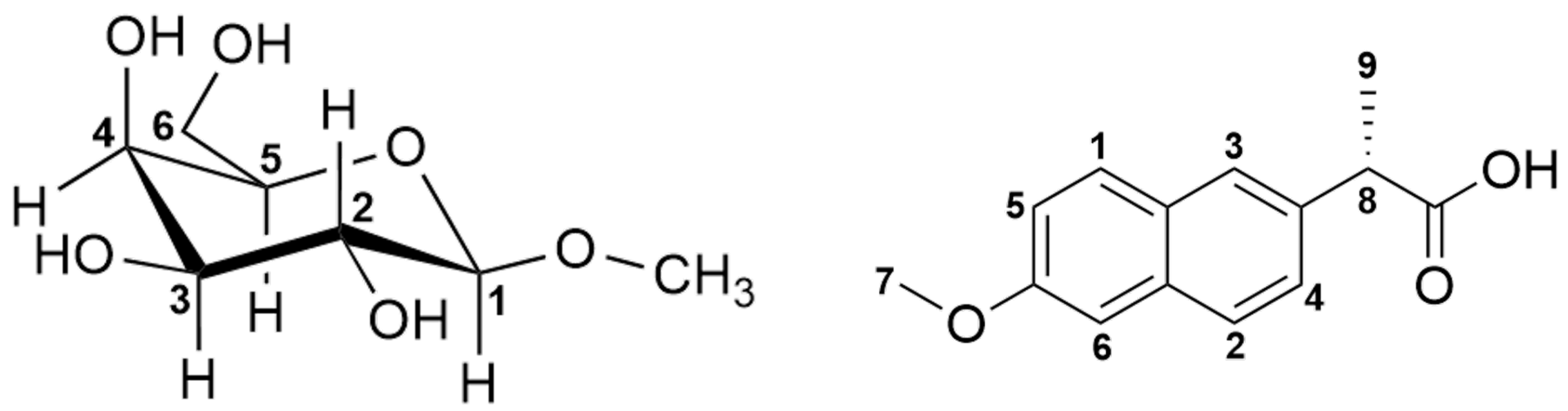

Figure S1: Chemical structures of methyl-β-D-galactoside (left) and Naproxen (right). Numbering is indicated with labels next to each carbon atom.

3. Dissociation constants (K_Ds)

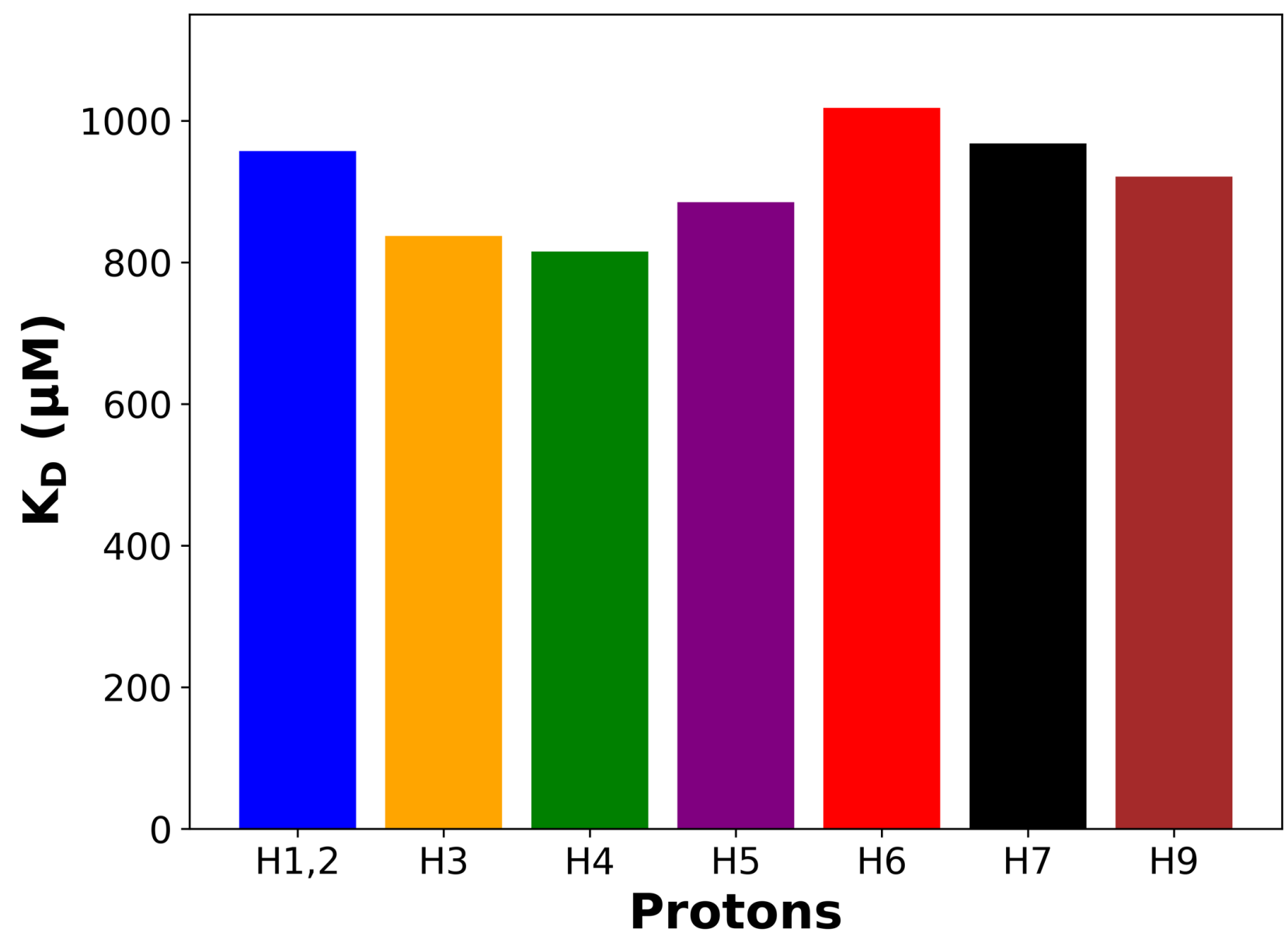

Figure S2: K_D values obtained for each proton of the BSA complex using the rd-STD NMR approach. 0.5 and 6 s were used as short and long saturation times, respectively.

4. STD NMR build-up curves

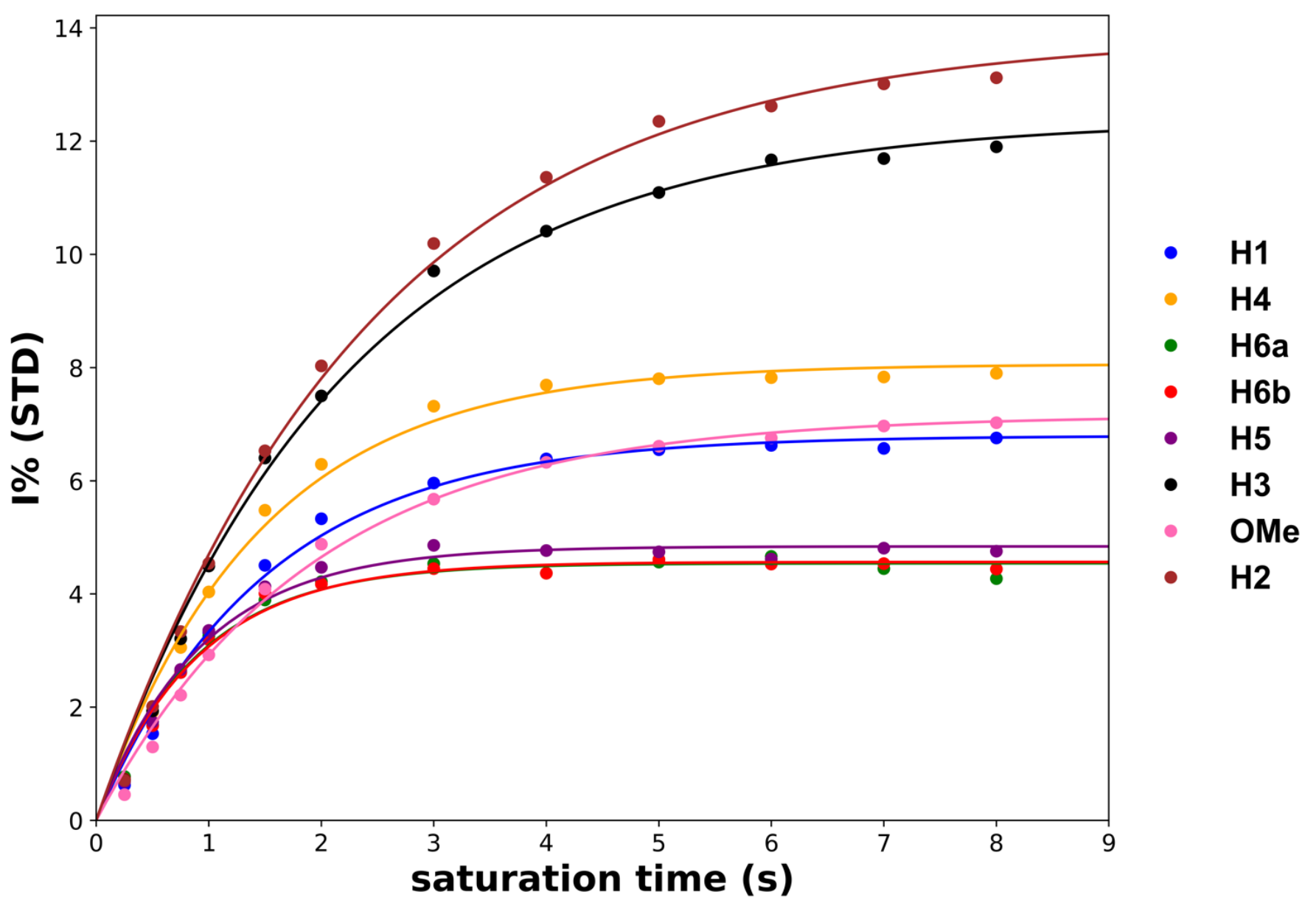

Figure S3: Experimental STD NMR build-up curves of RCA complex at 5 °C with irradiation at 0.66 ppm.

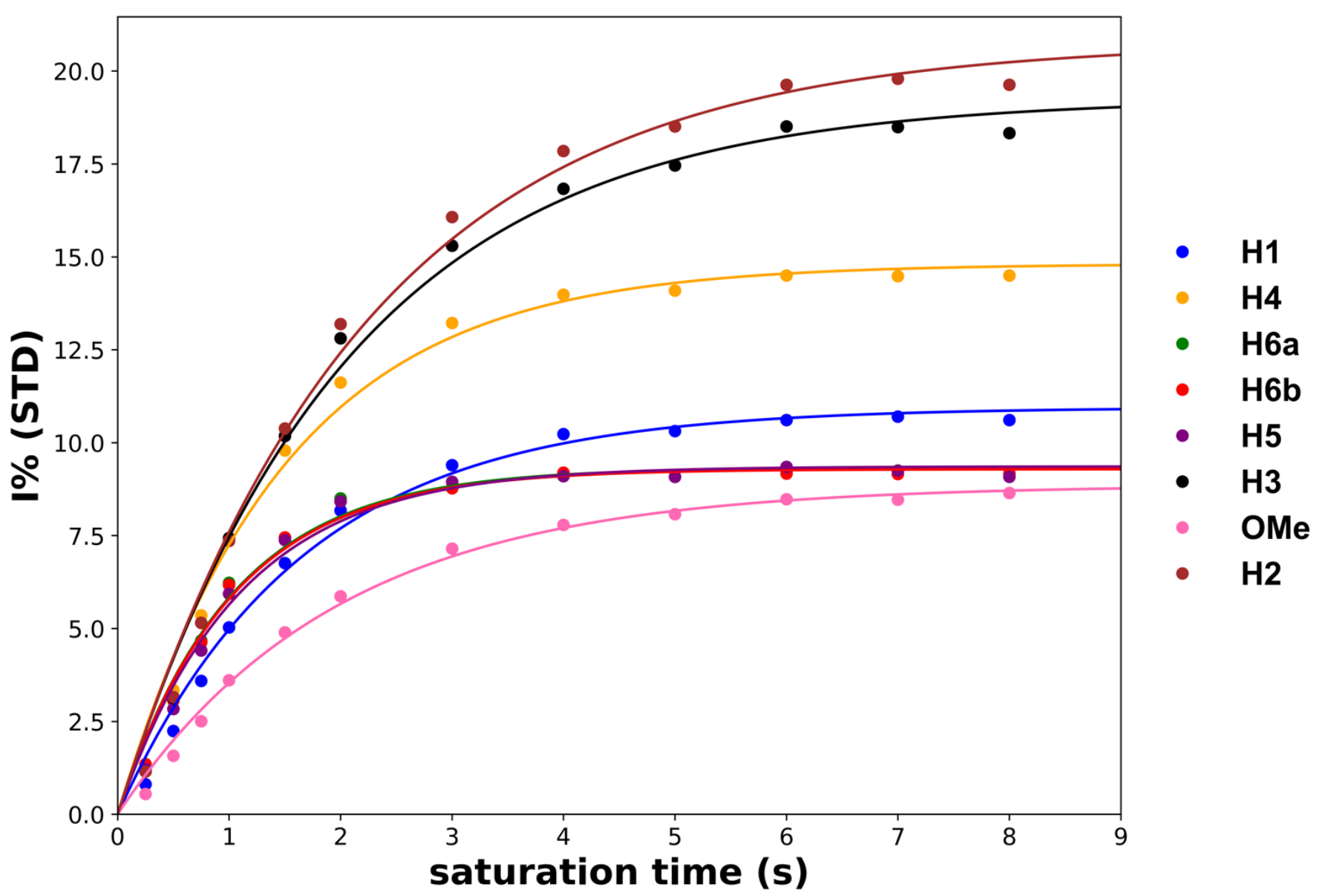

Figure S4: Experimental STD NMR build-up curves of RCA complex at 15 °C with irradiation at 0.80 ppm.

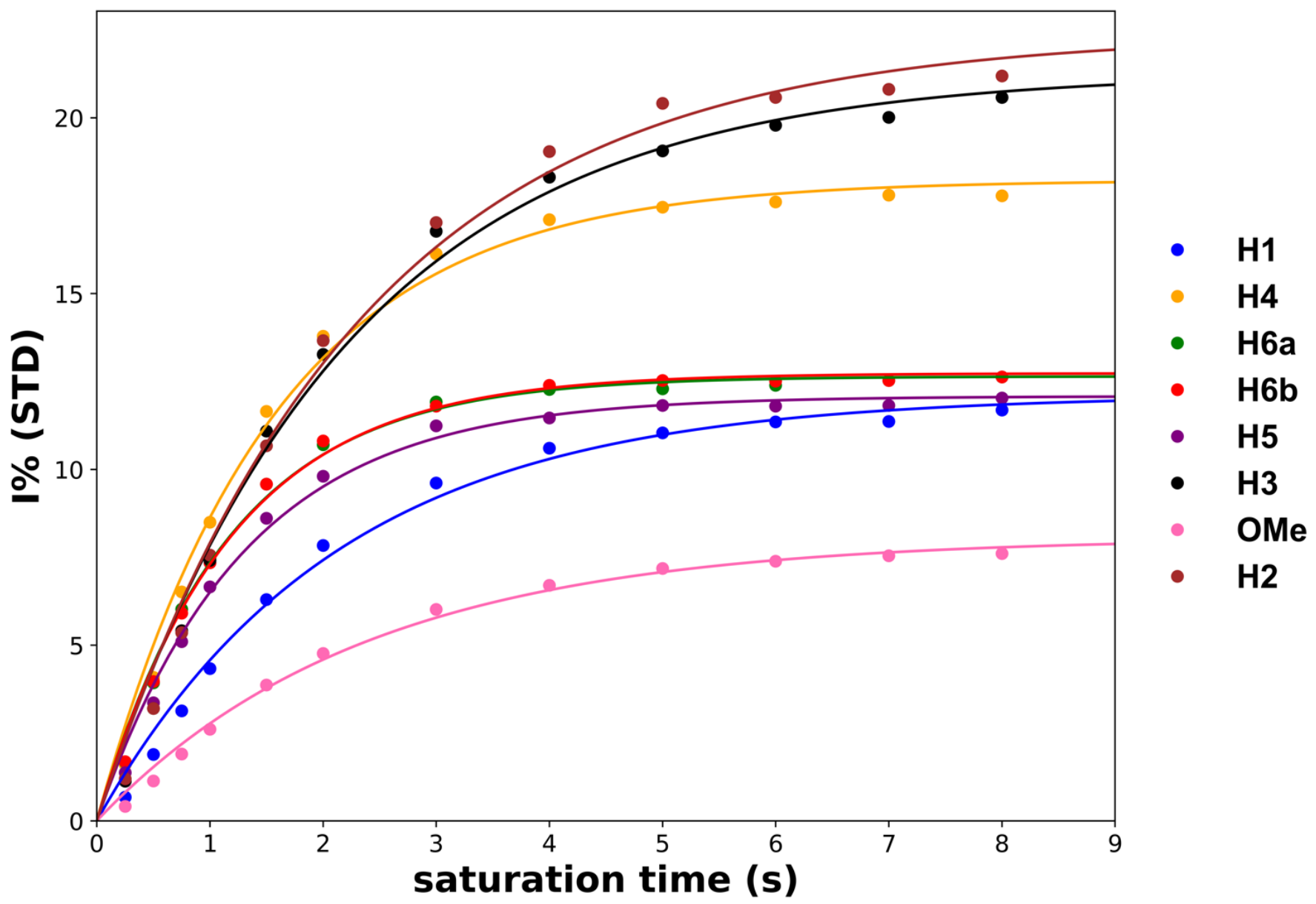

Figure S5: Experimental STD NMR build-up curves of RCA complex at 25 ºC with irradiation at 0.90 ppm.

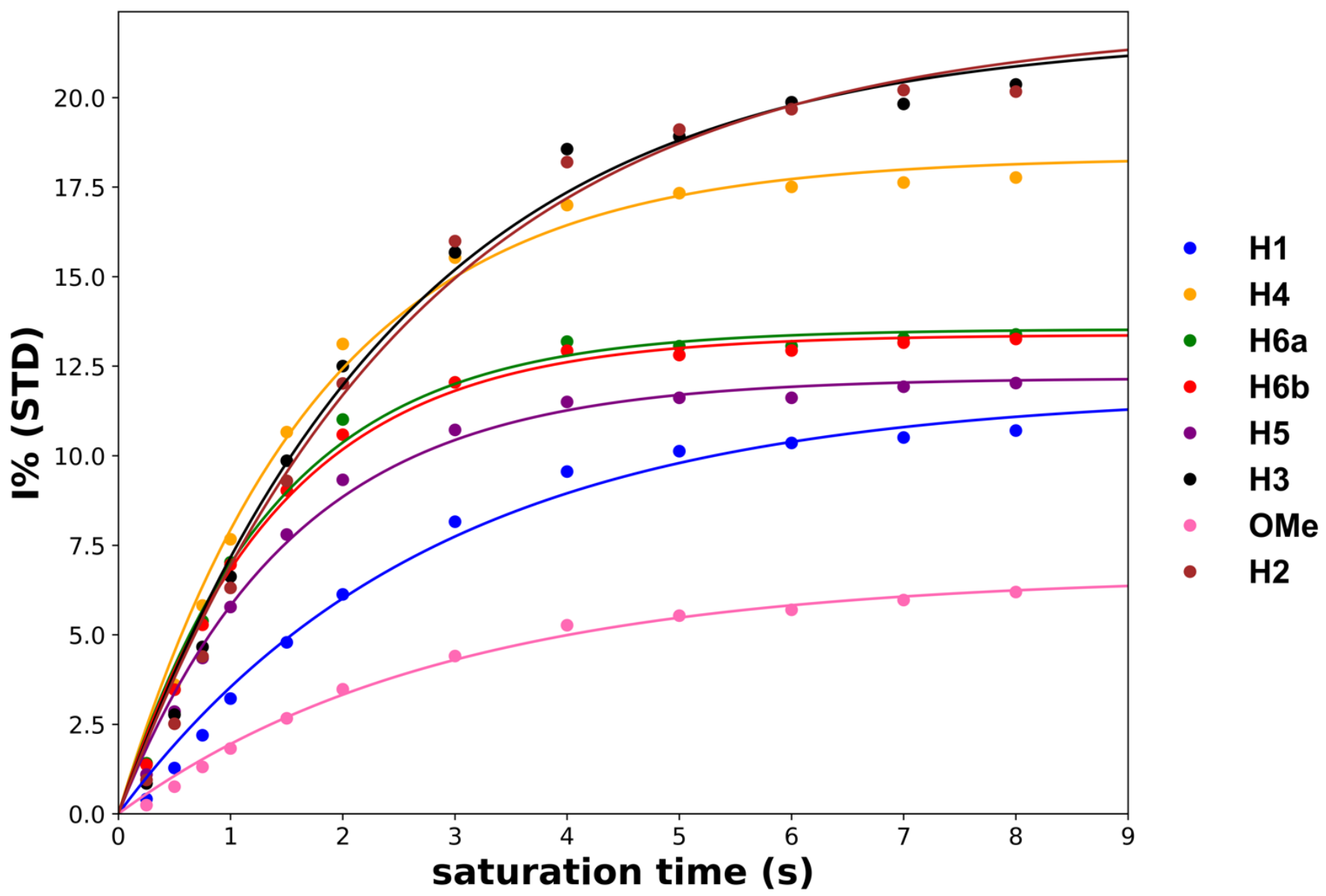

Figure S6: Experimental STD NMR build-up curves of RCA complex at 35 ºC with irradiation at 1.00 ppm.

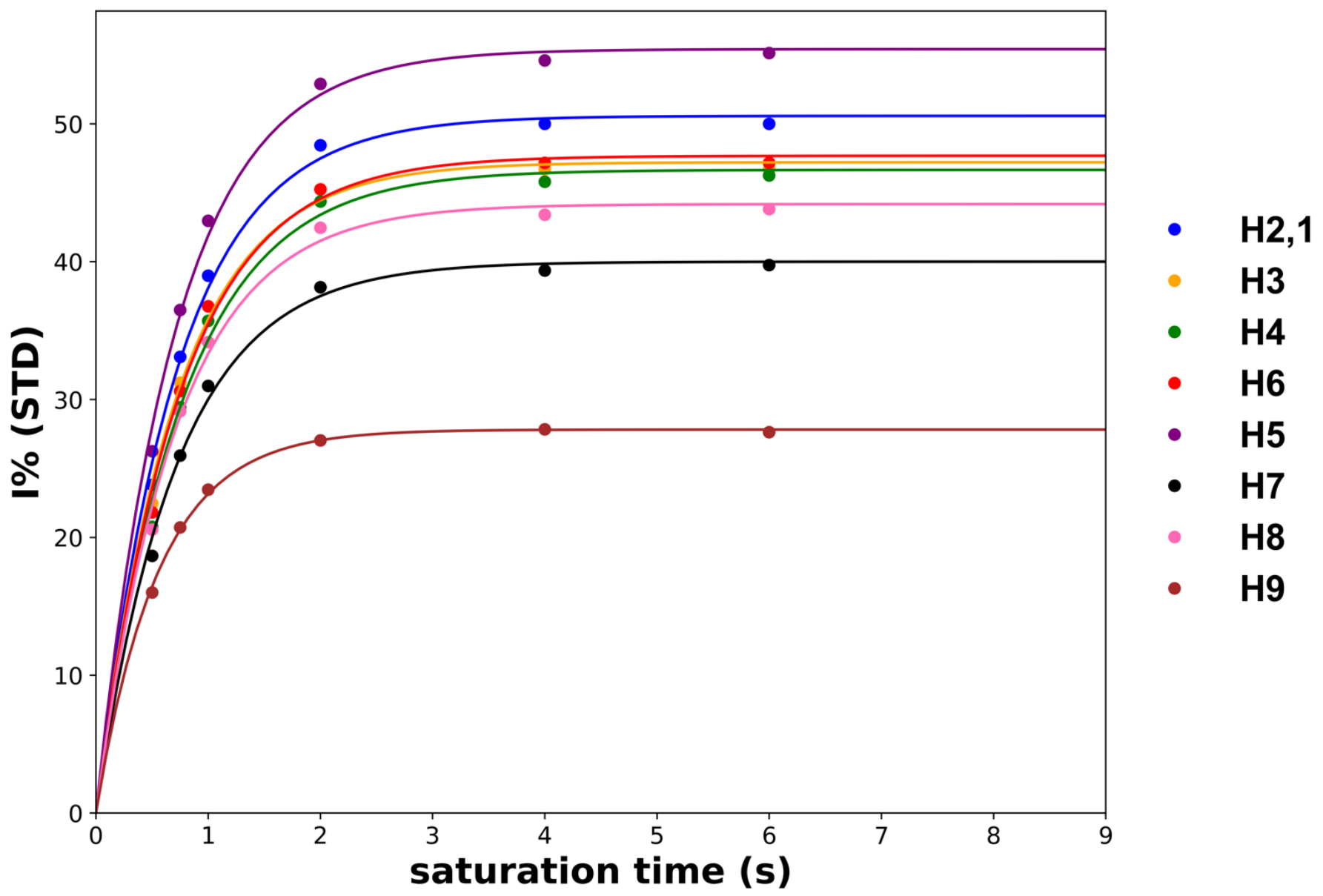

Figure S7: Experimental STD NMR build-up curves of BSA complex at 5 ºC with irradiation at 0.00 ppm.

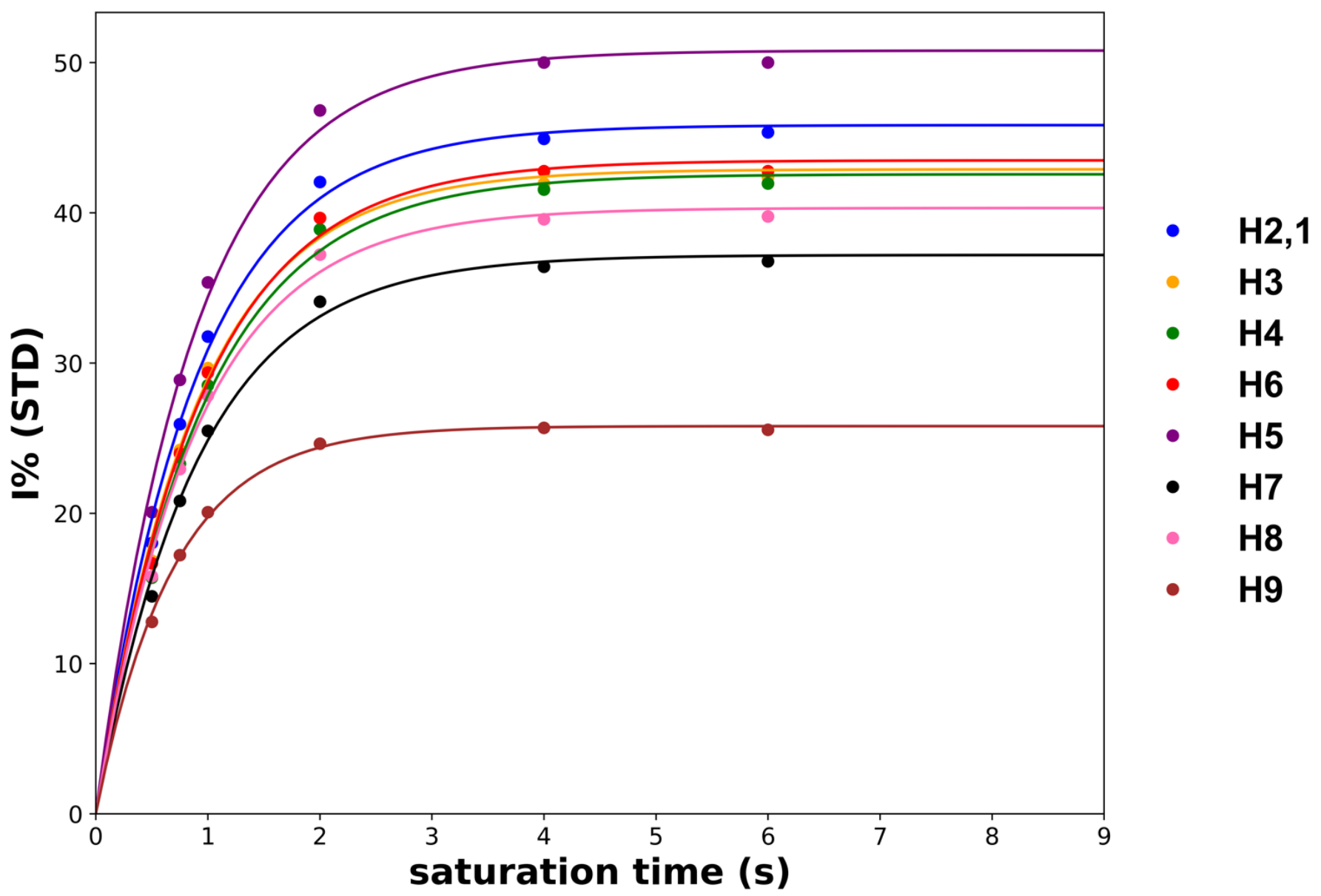

Figure S8: Experimental STD NMR build-up curves of BSA complex at 15 ºC with irradiation at 0.13 ppm.

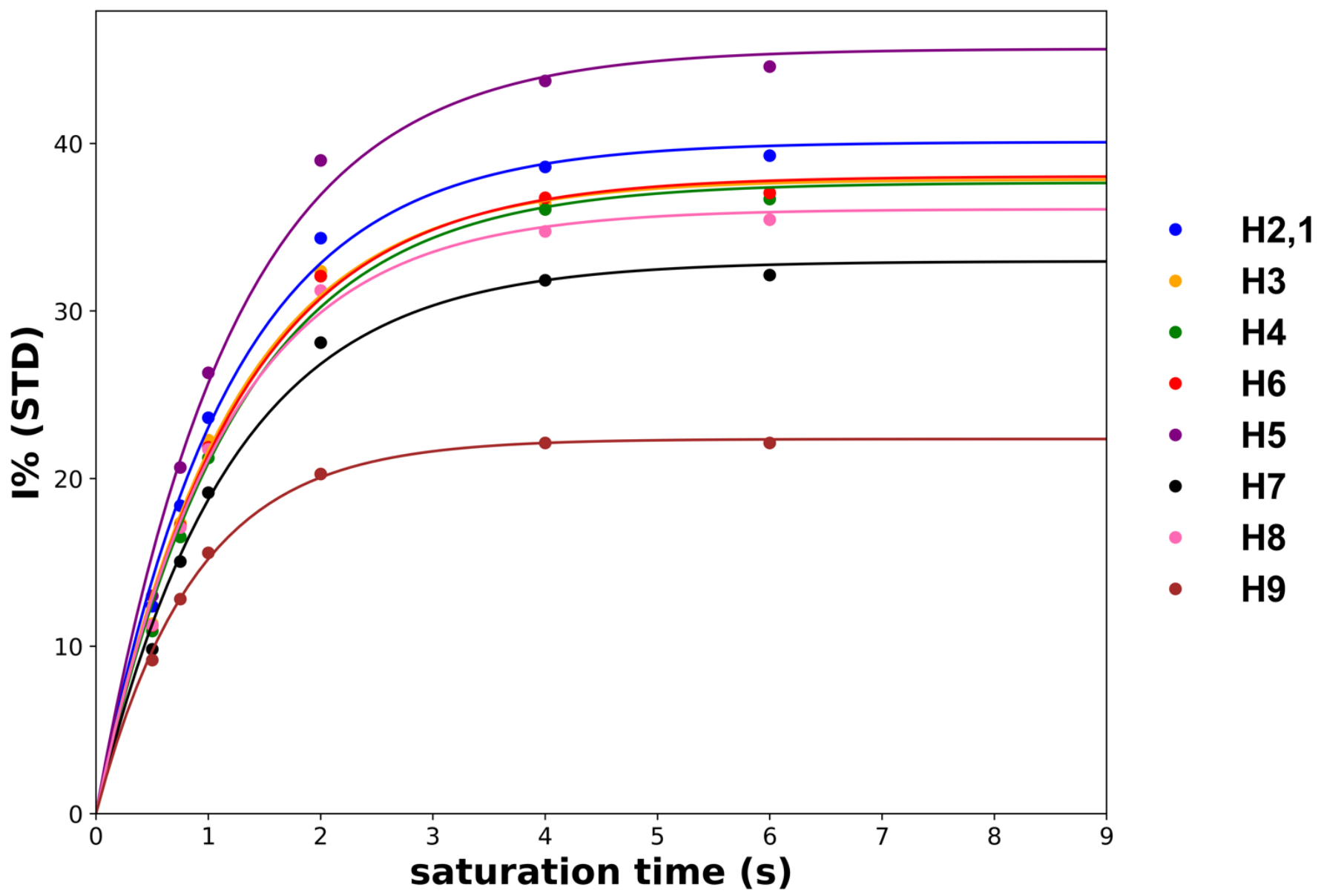

Figure S9: Experimental STD NMR build-up curves of BSA complex at 25 °C with irradiation at 0.25 ppm.

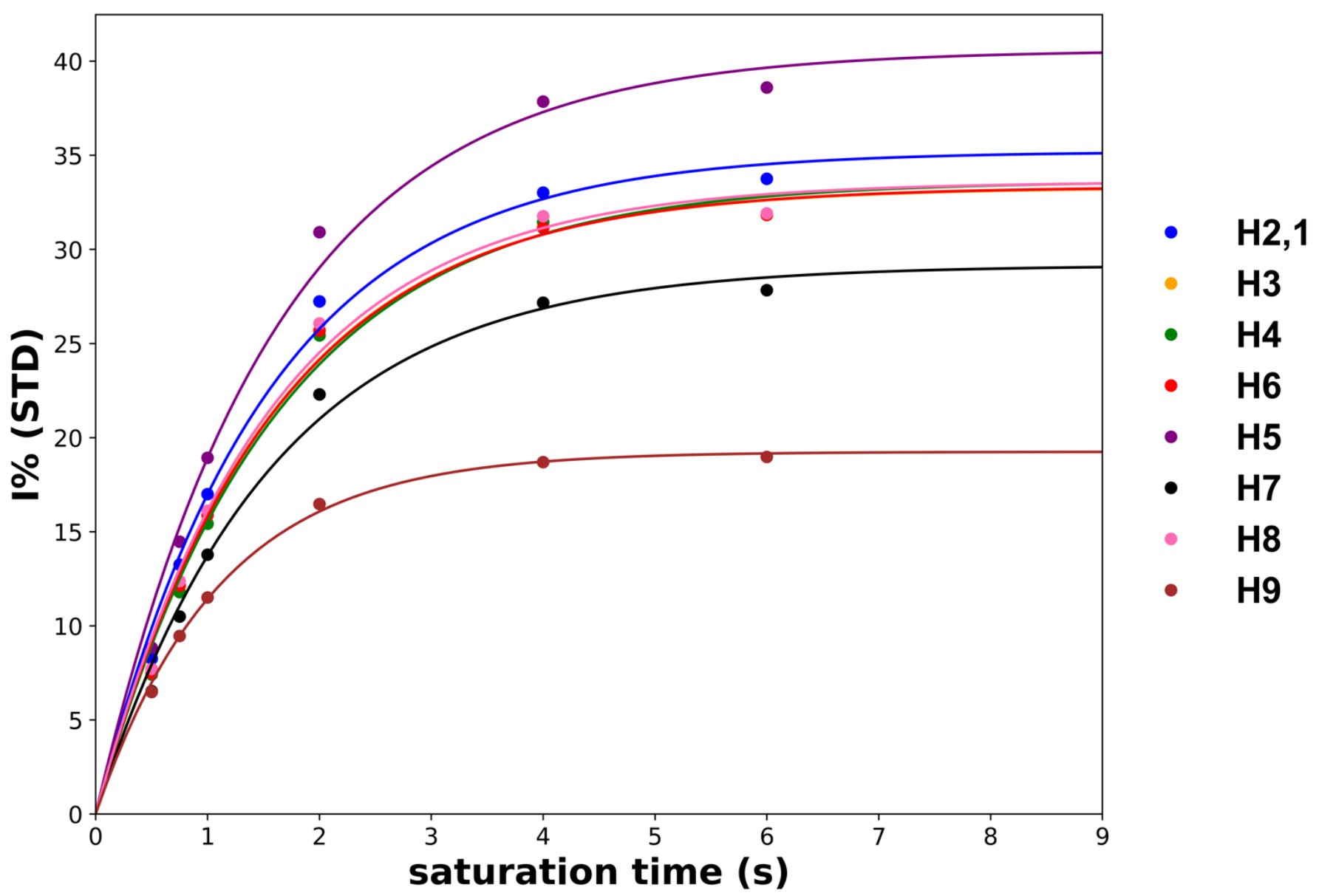

Figure S10: Experimental STD NMR build-up curves of BSA complex at 35 °C with irradiation at 0.36 ppm.
